# Mechanical Model for Catch-Bond-Mediated Cell Adhesion in Shear Flow

**DOI:** 10.3390/ijms21020584

**Published:** 2020-01-16

**Authors:** Long Li, Wei Kang, Jizeng Wang

**Affiliations:** 1 Key Laboratory of Mechanics on Disaster and Environment in Western China, Ministry of Education, College of Civil Engineering and Mechanics, Lanzhou University, Lanzhou 730000, China; kangw14@lzu.edu.cn; 2 PULS Group, Institute for Theoretical Physics, FAU Erlangen-Nürnberg, 91058 Erlangen, Germany

**Keywords:** catch bond, rolling adhesion, Markovian process, shear rate, flow-enhanced adhesion, bistability

## Abstract

Catch bond, whose lifetime increases with applied tensile force, can often mediate rolling adhesion of cells in a hydrodynamic environment. However, the mechanical mechanism governing the kinetics of rolling adhesion of cells through catch-bond under shear flow is not yet clear. In this study, a mechanical model is proposed for catch-bond-mediated cell adhesion in shear flow. The stochastic reaction of bond formation and dissociation is described as a Markovian process, whereas the dynamic motion of cells follows classical analytical mechanics. The steady state of cells significantly depends on the shear rate of flow. The upper and lower critical shear rates required for cell detachment and attachment are extracted, respectively. When the shear rate increases from the lower threshold to the upper threshold, cell rolling became slower and more regular, implying the flow-enhanced adhesion phenomenon. Our results suggest that this flow-enhanced stability of rolling adhesion is attributed to the competition between stochastic reactions of bonds and dynamics of cell rolling, instead of force lengthening the lifetime of catch bonds, thereby challenging the current view in understanding the mechanism behind this flow-enhanced adhesion phenomenon. Moreover, the loading history of flow defining bistability of cell adhesion in shear flow is predicted. These theoretical predictions are verified by Monte Carlo simulations and are related to the experimental observations reported in literature.

## 1. Introduction

Cell adhering to one another or their extracellular matrix under hydrodynamic flow is a crucial issue in many basic physiological and pathological processes, such as immune response [1,2], tumor metastasis [3], and targeted delivery of therapeutics to tissues [4]. For example, in leukocyte extravasation, the circulating leukocytes are recruited to the damaged or inflamed tissues with blood flow. In this case, the successful arrest and attachment of rolling leukocytes to vascular endothelium require interaction between membrane receptors and their ligands on the endothelial surface to sufficiently withstand the applied force from blood flow. Referring to this ability of specific recognition of rolling leukocytes, artificial nanocarriers with adhesive properties of leukocytes can be treated as potential platforms for targeted drug delivery in blood circulation [5]. Therefore, fully understanding the rolling adhesion of cells in a fluid environment will contribute greatly to fundamental biological processes and biomedical applications.

Cell adhesion is essentially mediated by the binding of receptors and ligand molecules. In principle, the formation and dissociation of receptor-ligand bonds are reversible and involve stochastic chemical reactions. Particularly, for traditional receptor-ligand bonds (slip bonds), the lifetime decreases with tensile force applied to the bond [6]. To explain the mechanism of this bond dissociation process under loading force, the theoretical framework describing the kinetic reactions between receptors and ligands was initially established by Bell [7,8]. Nevertheless, there is a counterintuitive observation that one type of bonds (so-called catch bonds) can live longer at higher tensile force [9,10]. Subsequently, two-pathway models of catch bond have been proposed to describe the kinetic reaction of catch-bond dissociation [11,12,13]. In addition to the chemical reactions of receptor–ligand bonds, the effect of other physical and mechanical factors, such as loading condition [14,15,16,17], deformation of substrate [18,19,20,21,22,23,24,25], membrane fluctuation [26,27,28,29], multiple bonds [30,31], cluster size [32], and cytoskeletal contraction [33,34] on cell or membrane adhesion have been discussed extensively. However, these studies focus on the occurrence of adhesion in a relatively quiescent environment.

Although plenty of experimental and theoretical efforts [35,36,37,38,39,40,41,42,43,44,45] have been conducted to elucidate the kinetic and stochastic behaviors of rolling adhesion via slip bonds in response to stimuli from shear flow, relatively few studies have investigated catch-bond-mediated adhesion dynamics under shear flow. The adhesion of leukocytes through selectin–ligand bond (a typical kind of catch bond) has been experimentally proposed to exhibit the shear threshold effect, in which increasing levels of shear rate stabilize leukocyte rolling under flow until the threshold of shear rate [9,46,47,48]. A number of adhesive dynamics simulations [49,50,51] have suggested that the catch behavior of bonds may result in this shear threshold phenomenon. However, this suggestion was not directly confirmed. A theoretical model that can extract the dynamics of cell rolling along substrate via catch bonds in shear flow and examine the mechanical mechanism underlying such shear threshold phenomenon is needed. In the present paper, we extend our previous adhesion model to establish a mechanical description based on energy conservation in cell adhesion by taking into account the stochastic reaction of catch bonds as well as hydrodynamic impact from shear flow.

## 2. Theoretical Model

**Figure 1:**
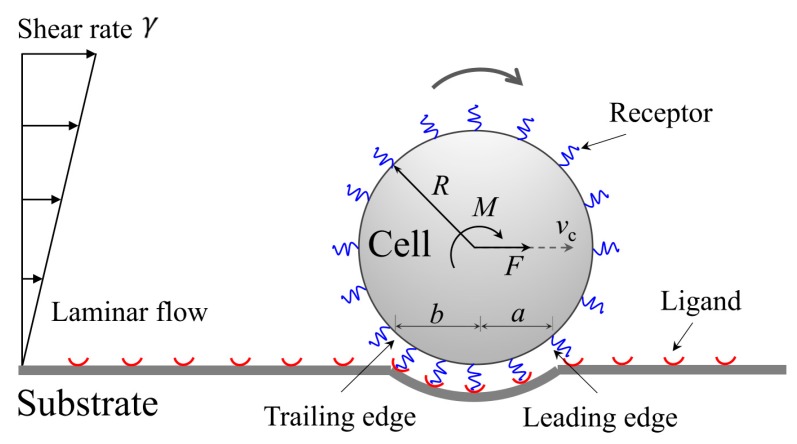
Schematic of rolling adhesion of cell to substrate under shear flow.

Figure 1 schematically illustrates a rolling cell in adhesive interaction with an elastic substrate via catch-bond cluster under laminar flow of shear rate γ. To describe the problem conveniently, we treat the cell as a rigid circular cylinder of radius *R* (two-dimensional problem). Parameters *a* and *b* represent the distances from the right and left contact edges to the apex of contact, respectively. The catch bonds are represented by thermalized harmonic springs with elastic stiffness $k_{\mathrm{LR}}$ and rest length $l_{b}$. The flow shear stress imposes a propulsive force *F* and a driving torque *M* to the rolling cell. The cell is then dragged by the tether to substrate through bonds due to trailing edge separation.

### 2.1. Lifetime of Catch Bonds

**Figure 2:**
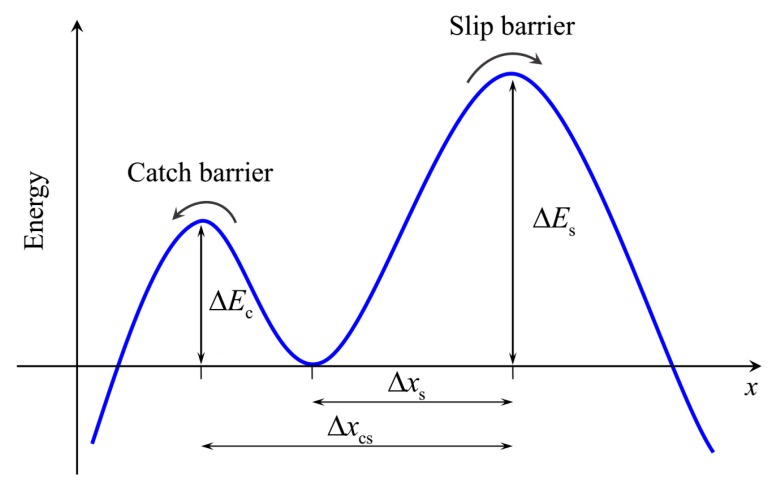
Schematic of conceptual energy landscape of two-pathway model [11,12]. A Brownian virtual particle can either escape over the catch barrier or over the slip barrier, starting from the bottom of the potential well.

We first consider the adhesion of cell and substrate under interface separation to examine the lifetime of catch bonds near the trailing edge. Hence, the stochastic process of the adhesion cluster of catch bond can be assumed as a Markov process, as governed by the master equation [52]:

(1) $$\frac{dp_{n}}{dt}=\left(n+1\right)k_{\mathrm{off}}p_{n+1}+\left[N_{t}-\left(n-1\right)\right]k_{\mathrm{on}}p_{n-1}-\left[nk_{\mathrm{off}}+\left(N_{t}-n\right)k_{\mathrm{on}}\right]p_{n},$$

where $p_{n}$ is the probability that *n* bonds are closed at time *t*. The total number of bonds near the trailing edge is denoted as $N_{t}$. Considering the dissociation process of a single catch bond as a Brownian virtual particle escaping out of the potential well via two alternative pathways, as shown in Figure 2, Evans et al. [11] proposed a two-pathway model to determine the dissociation rate of a catch bond by

(2) $$k_{\mathrm{off}}=\frac{\Phi_{0}k^{c}+\exp\left(f/f_{\mathrm{cs}}\right)\left[k^{s}\exp\left(f/f_{\beta }\right)\right]}{\Phi_{0}+\exp\left(f/f_{\mathrm{cs}}\right)},$$

where *k*^c^ and *k*^s^ are the spontaneous dissociation rates in the absence of a loading force *f* through the catch and slip pathways, respectively; and $f_{\beta }\equiv k_{B}T/\Delta x_{s}$ with the width from the bottom to the slip barrier of the potential well, $\Delta x_{s}$, and $f_{\mathrm{cs}}\equiv k_{B}T/\Delta x_{\mathrm{cs}}$ with the interval between two barriers, $\Delta x_{\mathrm{cs}}$, are the two characteristic force scales, respectively. In an unstressed condition, $\Phi_{0}=\exp\left[\left(\Delta E_{s}-\Delta E_{c}\right)/k_{B}T\right]$ is the equilibrium constant between the two pathways by Boltzmann distribution with two related energy barriers, $\Delta E_{s}$ and $\Delta E_{c}$. For typical catch bonds, the catch barrier $\Delta E_{c}$ is lower than the slip barrier $\Delta E_{s}$. However, in the limit of $\Delta E_{c}\gg \Delta E_{s}$, Equation (2) can completely be reduced to the Bell rate that describes a slip bond breaking [7,8], indicating the rupture of bond only through the slip pathway. Given the intersurface separation between cell and substrate, δ, the loading force experienced by each closed catch bond, *f*, can also be expressed as $f=k_{\mathrm{LR}}\left(\delta -l_{b}-l_{\mathrm{bind}}\right)$. The related total force, $f_{t}$, has a form of $f_{t}=f\sum_{n=0}^{N_{t}}np_{n}$. The sum term in the above equation is the average number of closed bonds.

In Equation (1), the association rate, $k_{\mathrm{on}}$, of bonds can be given by [53]

(3) $$k_{\mathrm{on}}=\frac{k_{\mathrm{on}}^{0}}{Z}\exp\left[-\frac{k_{\mathrm{LR}}{\left(\delta -l_{b}-l_{\mathrm{bind}}\right)}^{2}}{2k_{B}T}\right]$$

where $l_{\mathrm{bind}}$ is the reaction radius around the binding site, $k_{\mathrm{on}}^{0}$ is the spontaneous association rate, and the partition function *Z* can be determined by [19]

(4) $$Z=\frac{1}{l_{\mathrm{bind}}}\sqrt{\frac{\pi k_{B}T}{2k_{\mathrm{LR}}}}\left[\mathrm{erf}\left(\left(\delta -l_{b}\right)\sqrt{\frac{k_{\mathrm{LR}}}{2k_{B}T}}\right)+\mathrm{erf}\left(l_{b}\sqrt{\frac{k_{\mathrm{LR}}}{2k_{B}T}}\right)\right]$$

With the error function, erf (·).

**Figure 3:**
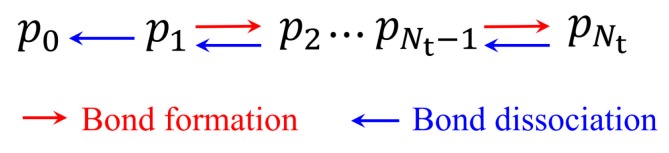
Kinetic network describing the evolution of the number of closed bonds.

The lifetime of catch-bond cluster can be defined by an average timescale to reach rupture of all bonds, starting from a cluster consisting of $N_{t}$ bonds. Therefore, we consider such stochastic process of bond cluster as a transition state problem, as illustrated in Figure 3. We can then solve the master equation, Equation (1), by using conventional numerical methods under reflecting and absorbing boundary conditions at the states of n=0 and $n=N_{t}$, respectively, as shown in Figure 3. Subsequently, on the basis of the concept of mean first passage time [54], the probability density of lifetime of catch bonds, P(t), evolving with time can be obtained by

(5) $$P\left(t\right)=-\sum_{n=0}^{N_{t}-1}\frac{dp_{n}\left(t\right)}{dt}$$

The mean lifetime, τ, can hence be expressed by

(6) $$\tau =\int_{0}^{\infty }P\left(t\right)tdt.$$

After addressing the lifetime of catch-bond cluster near the trailing edge, the tether bonds inducing effective works of adhesion will be predicted in the following subsection.

### 2.2. Effective Works of Adhesion for Cell Adhesion

As the cell rolls along the surface of the substrate, the works of adhesion via bonds resist to its rolling and should be consumed by the shear flow. These works have significant relationships with the evolution of closed bonds. Specifically, with the rolling of the cell, the catch bonds would be initially formed near the leading edge, then maintain stochastic association and dissociation reactions within the contact region, and would be totally broken in the end during trailing edge separation.

After averaging the master equation (Equation (1)) over time [55], the Bell equation [7,8] governing the evolution of the average density of closed bond, ξ, can be yielded as

(7) $$\frac{d\xi }{dt}=k_{\mathrm{on}}\xi_{\mathrm{all}}-\left(k_{\mathrm{on}}+k_{\mathrm{off}}\right)\xi$$

With the density of total bonds, $\xi_{\mathrm{all}}$.

When the cell surface approaches to the substrate in the vicinity outside the leading edge, the intersurface separation is assumed to evolve through $\delta \left(t\right)=\delta_{0}-\frac{a}{R}v_{c}t$. Here, $v_{c}$ denotes the linear speed of the cell center during rolling, and the initial separation between two approaching surfaces, $\delta_{0}$, meets $\delta_{0}\gg l_{b}$. Once the closed bond density near the leading edge, $\xi_{\mathrm{LD}}\left(t\right)$, has been obtained by solving the governing equation, Equation (7), under initial condition, $\xi_{\mathrm{LD}}\left(0\right)=0$, the adhesive traction, referring to the mean force per unit contacting area, can be given by

(8) $$\sigma_{\mathrm{LD}}\left(t\right)=k_{\mathrm{LR}}\xi_{\mathrm{LD}}\left(t\right)\left(\delta_{0}-\frac{a}{R}v_{c}t-l_{b}-l_{\mathrm{bind}}\right)$$

The last right bracket term stands for the deformation of each closed bond. By considering a/R≈0.05 [35], the relevant advancing work of adhesion, $w_{a}$, can be determined by

(9) $$w_{a}\approx 0.05v_{c}\int_{0}^{t_{s}}\sigma_{\mathrm{LD}}\left(t\right)dt$$

In Equation (9), $t_{s}$ represents the time that the intersurface separation starts from initial $\delta_{0}$ to the value at the leading edge. Considering the fact that the bonds are at their unstressed length in the contact region, we can obtain $t_{s}=\left(\delta_{0}-l_{b}-l_{\mathrm{bind}}\right)/\left(0.05v_{c}\right)$.

During trailing edge separation with speed of $v_{c}b/R$ (in the normal direction), the elastic deformations of elongated bonds also contribute to the works of adhesion between cell and substrate. Taking t=0 as the moment of time when cell and substrate surfaces near the trailing edge start to separate, the associated separation can be given as a function of time, $\delta \left(t\right)=l_{b}+l_{\mathrm{bind}}+\frac{b}{R}v_{c}t$. Here, we assume b/R≈0.05. The closed bond density as a function of time near the trailing edge, $\xi_{\mathrm{TR}}\left(t\right)$, can be obtained by solving the governing equation (Equation (7)) under the initial condition [35]:

(10) $$\begin{array}{ll} \xi_{\mathrm{TR}}\left(0\right)= & \xi_{\mathrm{LD}|t=t_{s}}+\left(\frac{k_{\mathrm{on}|\delta =l_{b}+l_{\mathrm{bind}}}\xi_{\mathrm{all}}}{k_{\mathrm{on}|\delta =l_{b}+l_{\mathrm{bind}}}+k_{\mathrm{off}|\delta =l_{b}+l_{\mathrm{bind}}}}-\xi_{\mathrm{LD}|t=t_{s}}\right)\\ & \times \left\{1-\exp\left[-\left(k_{\mathrm{on}|\delta =l_{b}+l_{\mathrm{bind}}}+k_{\mathrm{off}|\delta =l_{b}+l_{\mathrm{bind}}}\right)\left(a+b\right)/v_{c}\right]\right\} \end{array}$$

Here, the initial value $\xi_{\mathrm{TR}}\left(0\right)$ accounts for the evolution of closed bond density from the onset of the attachment near the leading edge to the start of the detachment at the trailing edge (see our previous work [35] for details).

The adhesive force per unit area and the effective receding work of adhesion can be defined by

(11) $$\sigma_{\mathrm{TR}}\left(t\right)\approx 0.05v_{c}tk_{\mathrm{LR}}\xi_{\mathrm{TR}}\left(t\right)\;\mathrm{and}\;w_{r}\approx 0.05v_{c}\int_{0}^{t_{c}}\sigma_{\mathrm{TR}}\left(t\right)dt$$

where $t_{c}$ means the cutoff time when most (99.9999% considered here) bonds are broken. As the cell velocity, $v_{c}$, tends to zero, the association and dissociation reactions of bonds reach equilibrium in every moment, and hence there is $w_{r}=w_{a}=w^{\mathrm{QS}}$ with the value, $w^{\mathrm{QS}}$, in a quasi-static process.

The effective advancing and receding works of adhesion can be numerically calculated from Equations (9) and (11). On the basis of the calculated works of adhesion, the kinetics of cell rolling will be addressed by taking into account energy conservation in cell rolling.

### 2.3. Kinetics of Cell Rolling in Shear Flow

According to the basic fluid mechanics [56], the propulsive force and driving torque per unit thickness imposed on a stationary rigid cylinder are 4πηRγ and $2\pi \eta R^{2}\gamma$, respectively, with the viscosity of flow, η. Here, following our previous work [35], we assume the hydrodynamic loadings exerted on a rolling cell as

(12) $$F\left(t\right)=4\pi \eta R\left[\gamma -v_{c}\left(t\right)/R\right]\;\mathrm{and}\;M\left(t\right)=2\pi \eta R^{2}\left[\gamma -v_{c}\left(t\right)/R\right]$$

The work done by the shear flow from time 0 to *t* can be readily expressed as

(13) $$U_{1}\left(t\right)=\int_{0}^{t}F\left(t\right)v_{c}\left(t\right)dt+\int_{0}^{t}M\left(t\right)\omega \left(t\right)dt$$

where $\omega \left(t\right)=v_{c}\left(t\right)/R$ is the angular velocity of rolling cell, as shown in Figure 1. Additionally, the kinetic energy of cell is expressed by

(14) $$U_{2}\left(t\right)=\frac{1}{2}J_{0}\omega^{2}\left(t\right)$$

where $J_{0}=3mR^{2}/2$ denotes the rotational inertia of the cell concerning the apex of contact and $m=\pi \rho R^{2}$ is the cell mass with its material density, ρ.

By considering the equilibrium conditions for such an adhesion system, the resistant torque caused by adhesion with the substrate can be expressed by [57]

(15) $$M_{r}\left(t\right)=R\left[w_{r}\left(t\right)-w_{a}\left(t\right)\right]$$

The energy dissipation due to the hysteresis of cell adhesion can thus be given by

(16) $$U_{3}\left(t\right)=\int_{0}^{t}M_{r}\left(t\right)\omega \left(t\right)dt$$

According to energy conservation, a rolling cell with initial kinetic energy, $U_{2}\left(0\right)$, should have

(17) $$U_{1}\left(t\right)+U_{2}\left(0\right)=U_{2}\left(t\right)+U_{3}\left(t\right)$$

After substituting Equations (12–16) into Equation (17), taking the derivative with respect to time yields

(18) $$\frac{J_{0}}{R}\frac{dv_{c}\left(t\right)}{dt}=M\left(t\right)+F\left(t\right)R-\left[w_{r}\left(t\right)-w_{a}\left(t\right)\right]R$$

Equation (18) indeed describes the torque equilibrium of cell. The left term is the inertia moment. The right-hand side stands for the resultant torque applied on the cell. Inserting Equation (12) into Equation (18) by normalization manipulations gives the dynamic governing equation of cell rolling as

(19) $$\frac{d{\bar{v}}_{c}\left(\bar{t}\right)}{d\bar{t}}=4\pi \left[\bar{\gamma }-{\bar{v}}_{c}\left(\bar{t}\right)\right]-\frac{2}{3}\left[{\bar{w}}_{r}\left(\bar{t}\right)-{\bar{w}}_{a}\left(\bar{t}\right)\right]$$

With ${\bar{v}}_{c}\left(\bar{t}\right)\equiv \frac{\eta v_{c}\left(t\right)}{w^{\mathrm{QS}}}$, $\bar{t}\equiv \frac{\eta t}{m}$, $\bar{\gamma }\equiv \frac{\eta R}{w^{\mathrm{QS}}}\gamma$, ${\bar{w}}_{r}\equiv \frac{w_{r}}{w^{\mathrm{QS}}}$, and ${\bar{w}}_{a}\equiv \frac{w_{a}}{w^{\mathrm{QS}}}$. For a given initial cell speed, this governing equation can be numerically solved by the classic Runge-Kutta method. Hereafter, we take the representative values of system parameters as shown in Table 1.

## 3. Results and Discussion

### 3.1. Lifetime of Catch-Bond Cluster

**Figure 4:**
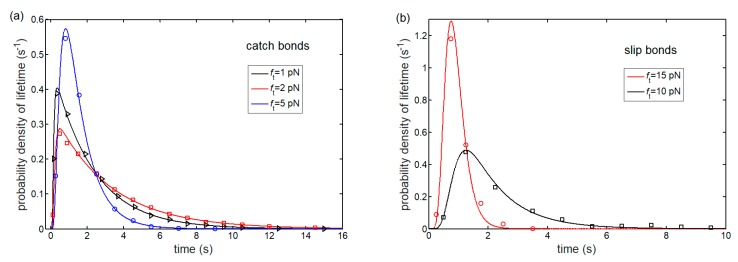
Probability density of lifetime of (**a**) catch-bond and (**b**) slip-bond clusters as a function time for $N_{t}=10$ and different interfacial forces (lines: theoretical predictions from Equation (5); symbols: Monte Carlo simulations).

To examine the catch-bond behavior, we begin by considering the relationship between lifetime of bond cluster and interfacial force during interface separation. Figure 4 plots the predicted probability distribution of cluster lifetime as a function of time under different interfacial forces for catch and slip bonds. As time goes on, the probability distribution of lifetime increases first, then decreases, and finally tends to zero for both catch and slip bonds. Likewise, the relevant Monte Carlo simulations (see Appendix A) are performed to validate the theoretical model. Comparison with our numerical results from Equation (5) shows excellent agreement (no fitting parameters).

**Figure 5:**
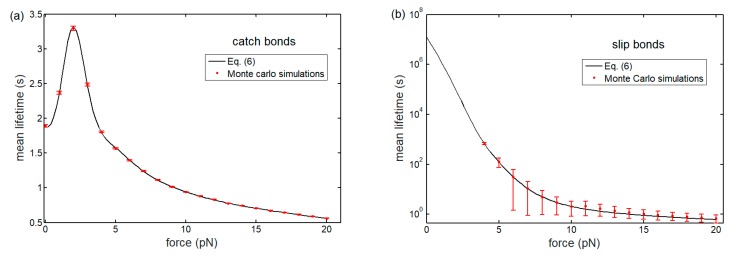
Mean lifetime of bond clusters as a function of interfacial force with $N_{t}=10$ for (**a**) catch and (**b**) slip bonds. The theoretical results (solid curves) are in good agreement with the simulations (discrete symbols).

The evolution of mean lifetime of bond clusters with the change of interfacial forces for catch and slip bonds is plotted in Figure 5. The mean lifetime of catch-bond cluster increases up to a maximum before decreasing with tensile force exerted on the bonds. This finding indicates that the catch bonds are initially strengthened by an increase in force. As expected, the mean lifetime of cluster monotonously decreases and eventually diminishes with respect to the force for slip bonds. However, although no catch behavior is observed at all for slip bonds, the slip bonds have much longer lifetime than that of catch bonds when the interfacial force is lower than 5 pN. As the slip barrier is stronger than the catch barrier, the slip bonds are more stable under low force. This distinction of force-dependent lifetime of single catch and slip bond is also observed in previous single-molecule force measurements [65].

**Figure 6:**
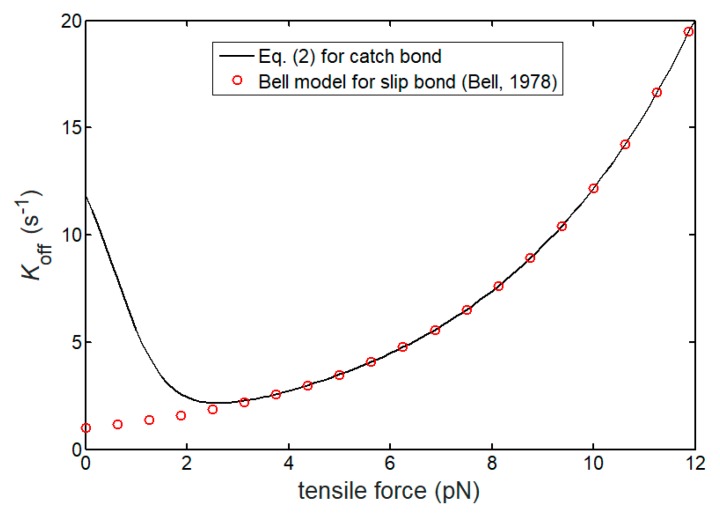
Dissociation rate of catch (**black line**) and slip (**red cycles**) bonds versus tensile force. The red cycles represent the theoretically predicted results for slip bond from the Bell model [7].

To understand the intrinsic mechanism resulting in these two different lifetimes between catch and slip bonds, Figure 6 shows a comparison of the dissociation rate between the two bonds as a function of the tensile force. There exists a critical tensile force corresponding to a minimum value of dissociation rate for catch bond. A single catch bond seems to maintain the most stable formation under this optimal tensile force. For a low tensile force, thermally induced escape of adhesion molecule from the weak catch barrier can easily make the catch bond break. If the tensile force is large, the force reducing the slip barrier height also destabilizes the catch bond, although the force simultaneously strengthens the catch barrier. Both low and large tensile forces result in short lifetime of catch bond. However, different from the catch bond, the dissociation rate of the slip bond monotonously increases as the tensile force increases owing to the gradually decreased slip barrier height by force, thereby leading to a force-reduced lifetime.

Once the force increasing the catch bonds’ lifetime (a characteristic feature of catch bonds) has been investigated, the works of adhesion via bonds resisting to the cell motion in shear flow will be shown in the following.

### 3.2. Works of Adhesion

**Figure 7:**
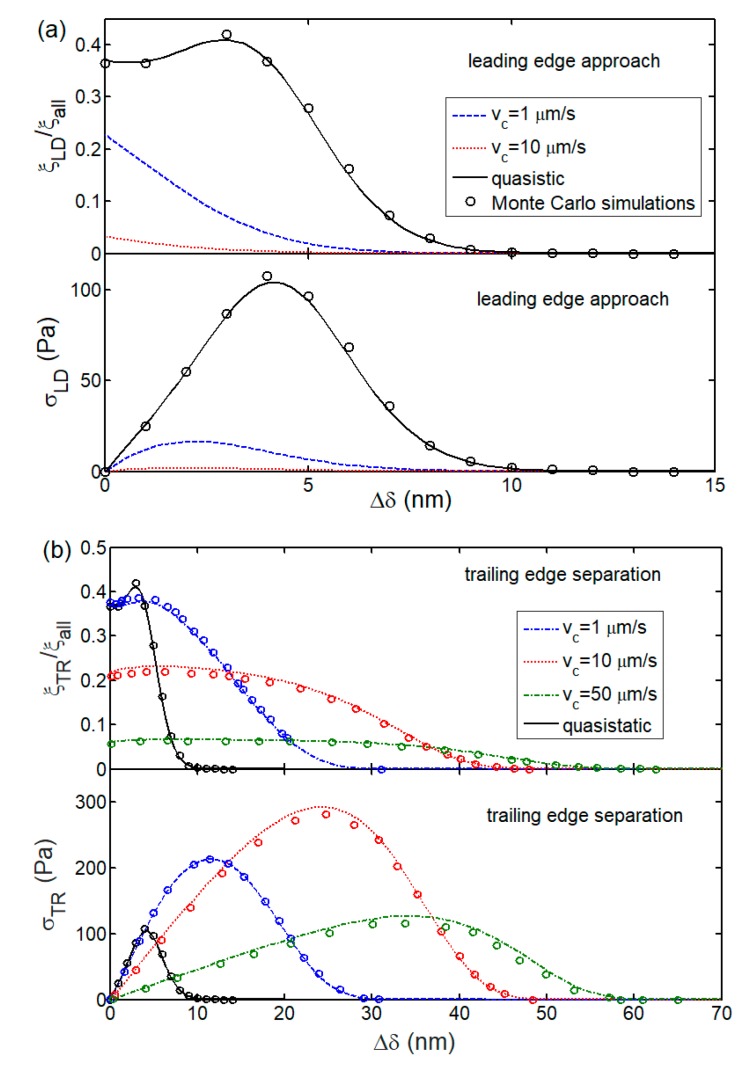
Normalized closed bond density and adhesion traction as a function of the change of intersurface separation ($\Delta \delta =\delta -l_{b}-l_{\mathrm{bind}}$) for (**a**) leading edge approach and (**b**) trailing edge separation processes by taking into account the different speeds of cell rolling. Curves are theoretical results and discrete symbols stand for results from Monte Carlo simulations.

In Figure 7, for a cell rolling along the substrate, we plot the variation of normalized closed bond density near the leading and trailing edges with the change of intersurface separation between the cell surface and substrate for different speeds of cell rolling. The closed bond density and adhesion traction show distinct dependences on the speed of cell rolling. During leading edge approach, the closed bond density monotonously increases as the change of separation decreases for $v_{c}=1\;\mathrm{and}\;10\;\mu m/s$. This is because of the domination of bond association in leading edge approach. However, in the limit of $v_{c}\to 0$ (quasi-static condition) with decreasing change of separation, the closed bond density, $\xi_{\mathrm{LD}}$, increases first, then reaches its maximum, and finally maintains within a relatively high range, as shown in Figure 7a. For the trailing edge separation, Figure 7b shows that the closed bond density first increases and then decreases to zero as the separation increases, implying a bond dissociation-induced fracture of cell-substrate interface.

In addition to closed bond density, Figure 7 also shows the evolution of related adhesion traction with the change of intersurface separation occurring near the leading and trailing edges. A critical separation associated with a maximum value of adhesion traction for both leading approach and trailing separation processes can be seen. This is a result of the competition between bond deformation and closed bond density.

**Figure 8:**
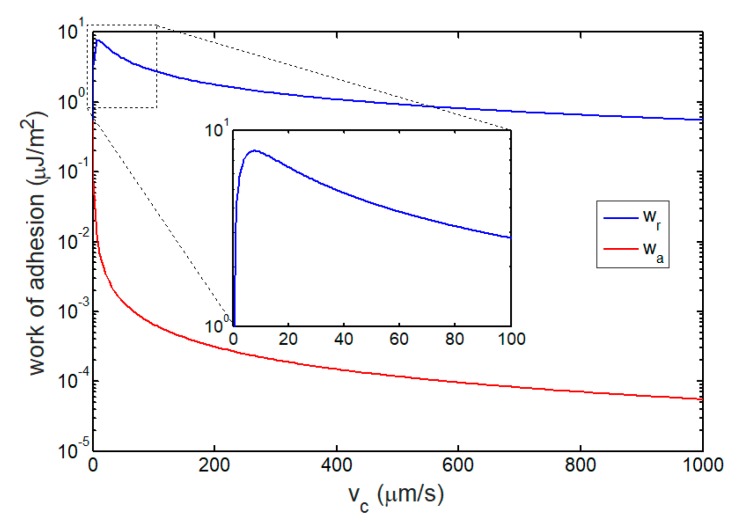
Work of adhesion as a function of cell speed. The inset is a partial enlarged drawing for the cell speed ranging from 0 μm/s to 100 μm/s. When $v_{c}\to 0$, the work of adhesion in quasi-static condition can be calculated as $w^{\mathrm{QS}}=0.54\;\mu J/m^{2}$.

After integrating the obtained adhesion traction with respect to the change of intersurface separation, Figure 8 shows the work of adhesion as a function of cell speed ranging from 0 μm/s to 1000 μm/s. As the cell speed increases, the advancing work of adhesion induced by leading edge approach, $w_{a}$, monotonously decreases from an initial value, $w^{\mathrm{QS}}$, because there is not enough time for bond formation at the high speed of approaching. However, for the receding work of adhesion, $w_{r}$, Figure 8 shows a maximum $w_{r}$ at a critical cell speed of $v^{*}\approx 7.5$ μm/s. This optimized work of adhesion can be explained by the competition between the stochastic reaction of molecular bonds and elongation of each closed bond. For a low cell speed, the stochastic reaction of molecular bonds dominates the evolution of closed bond density. Therefore, the closed bond under a large elongation cannot survive. When the cell speed is high, a small density of closed bond causes a low receding work of adhesion as there is not enough time to form bonds. The receding work of adhesion coupling closed bond density and bonds’ elongation would reach a maximum value at a critical cell speed.

### 3.3. Kinetics and Shear Threshold of Cell Rolling

**Figure 9:**
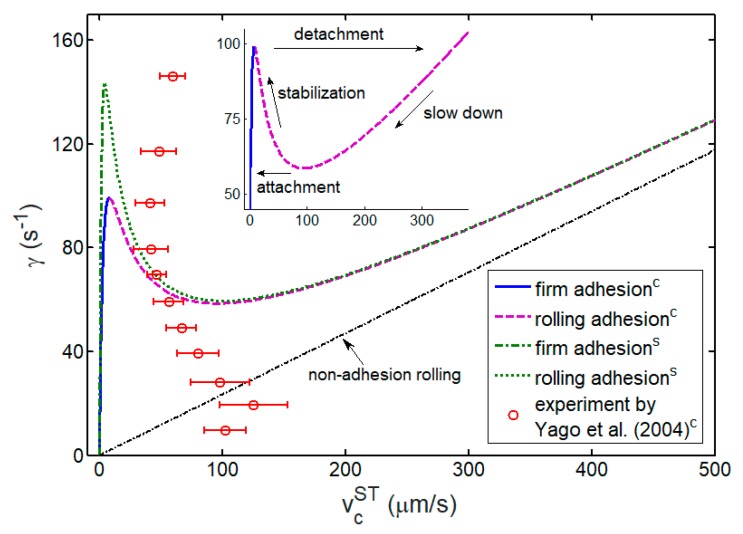
Steady speed of cell, $v_{c}^{\mathrm{ST}}$, as a function of shear rate of flow, *γ*. The superscripts c and s in the legend mark the catch-bond- and slip-bond-mediated cell adhesion, respectively. The blue solid and green dash–dot lines denote the firm adhesion state where the cell exhibits a slow motion. The purple dash and green dot lines represent the rolling adhesion state where the cell rolls along the substrate at a relatively high speed. The black dash–dot line means the non-adhesion rolling state, where the cell freely rolls without adhesion interaction between cell and substrate. The red circles are experimental results of neutrophils rolling on P-selectin glycoprotein ligand-1 (PSGL-1)-coated substrate under shear flow by Yago et al. [46], in which the P-selectin–PSGL-1 complex shows a typical catch behavior under tensile force. The inset is a partially enlarged drawing for the shear rate ranging from 45 s^−1^ to 105 s^−1^ and shows bistable processes of cell adhesion in a shear flow.

Once the advancing and receding works of adhesion have been calculated, as shown in Figure 8, the kinetics of cell rolling can be described on the basis of Equation (19). We consider the steady state of cell rolling in a shear flow by setting $d{\bar{v}}_{c}/d\bar{t}=0$. Subsequently, Figure 9 shows a comparison between theoretical predictions and experimental results by Yago et al. [46] for steady speed of a cell as a function of shear rate in the cell rolling process regulated by catch bonds. Two theoretically predicted critical shear rates for detachment and attachment can be seen for cell adhesion. Beyond the upper threshold, the shear flow keeps a cell rolling instead of firm adhesion states. Nevertheless, for the flow with a smaller shear rate than the lower threshold, such rolling adhesion of cell cannot happen. Particularly, with the shear rate ranging between these two critical shear rates, the cell is either at firm or at rolling adhesion that relies on the dynamic shear rate loading path of flow from initial zero shear rate (see Appendix B). This predicted bistable behavior of cell adhesion in shear flow is consistent with the previous experimental observations on leukocyte, circulating tumor cell, and red blood cell adhesion in shear flow [40,45,66,67]. Meanwhile, as the shear rate increases, the theoretically predicted steady cell speed is asymptotically close to the steady speed of cell free rolling, in which there is no adhesion between cell and substrate. This is because the faster the cell speed, the smaller the number of bonds formed during cell rolling.

Furthermore, Figure 9 shows that the cell steady speed decreases as the shear rate increases from lower to upper thresholds, indicating the ability of cells to withstand high shear stress better than low shear stress. The cell rolling begins to speed up as long as the unceasingly increased shear rate is larger than the upper thresholds. Such flow-enhanced cell adhesion phenomenon has also been observed in experiments [46,67,68,69], as shown in Figure 9. Here, we predict the upper threshold of shear rate for detachment as 99.2 s^−1^, which is close to the experimentally observed value range from 70 s^−1^ to 100 s^−1^ [46]. The quantitative differences between theoretical predictions (2D) and experimental observations (3D) are attributed to the distinction in 2D and 3D situations of cell rolling adhesion, especially for large shear rate.

Additionally, Figure 9 shows that the cell steady speed corresponding to the upper threshold of shear rate is 8 μm/s, which is close to $v^{*}$, a critical cell speed associated with a maximum receding work of adhesion, as shown in Figure 8. This result suggests that the flow-enhanced cell adhesion phenomenon can be explained by the maximum receding work of adhesion withstanding cell rolling. Nevertheless, extensive efforts have been made to treat the catch behavior of bond as the underlying mechanism of this flow-enhanced adhesion phenomenon [10,69,70,71]. To identify whether the catch behavior of bond dominates the flow-enhanced cell adhesion phenomenon, we also plot the predicted variation of steady cell speed with the shear rate for slip-bond-mediated cell rolling adhesion, as shown in Figure 8. The slip-bond-mediated cell adhesion clearly shows a similar flow-enhanced cell adhesion phenomenon, even though the slip bond does not exhibit the catch behavior at all, as shown in Figure 5. This finding directly indicates that the shear threshold phenomenon of cell rolling is regulated by the energy competition rather than the catch behavior of bond.

## 4. Conclusions

We have developed a mechanical model integrating hydrodynamic impact and stochastic process of formation and dissociation of bonds to investigate the dynamics of catch-bond-mediated cell adhesion in shear flow. Through energy conservation, the kinetic equation governing cell rolling under shear flow is obtained. Results showed that the shear rate of flow significantly affects the steady speed of cell rolling. Irrespective of the catch bond behavior of force-increased lifetime, shear-flow-stabilized cell adhesion occurred, which can be explained by the dynamic competition between kinetics of receptor-ligand reactions and cell rolling. Two critical shear rates are also identified to define the distinct steady states of cell adhesion, including firm, rolling, and bistable adhesion. These findings not only help us understand the mechanical mechanism of catch-bond-mediated rolling adhesion of cells in response to hydrodynamic impact but also can be useful in designing targeted therapy in biomedical applications.

## Figures and Tables

**Table 1:** Model parameters.

Quantity	Symbol	Value	Reference
Cell radius	*R*	4.25 μm	[58,59]
Cell density	ρ	1.1 g/cm^3^	[60]
Flow viscosity	η	0.001 Pa·s	[58]
Density of total bonds	$\xi_{\mathrm{all}}$	140 μm^−2^	[7,58]
Rest length of bond	$l_{b}$	11 nm	[19]
Reacting radius	$l_{\mathrm{bind}}$	1 nm	[61]
Bond stiffness	$k_{\mathrm{LR}}$	0.5 pN/nm	[62]
Spontaneous association rate	$k_{\mathrm{on}}^{0}$	25 s^−1^	[45,63]
Spontaneous dissociation rate of slip barrier	$k^{s}$	1 s^−1^	[61]
Spontaneous dissociation rate of catch barrier	$k^{c}$	15 s^−1^	[64]

**Figure A1:**
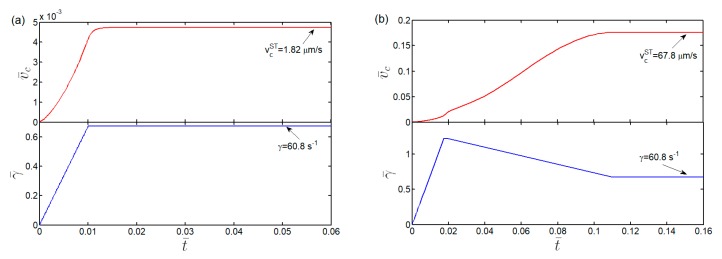

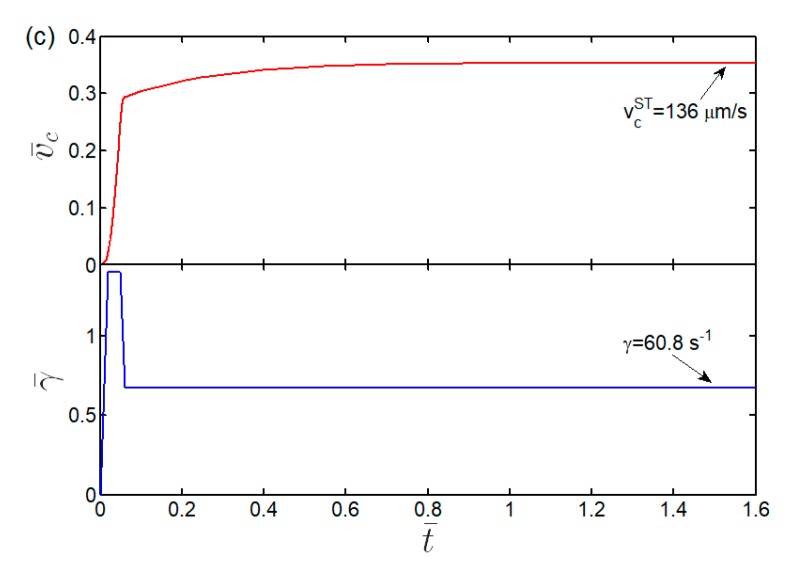
Dependence of steady speed of cell, $v_{c}^{\mathrm{ST}}$, on the shear rate loading history of flow. In the three types of loading cases above, the final steady shear rates of flow are the same and equal to 60.8 s^−1^. However, the corresponding steady speeds of cell are (**a**) 1.82, (**b**) 67.8, and (**c**) 136 μm/s.

## Appendix A. Monte Carlo Simulations

The stochastic processes of adhesion bonds described in Equations (1) and (7) can also be numerically obtained through Monte Carlo simulations. In light of the first reaction method derived from the Gillespie algorithm [72,73], the Monte Carlo simulations can be proposed using the following steps:

First, for each binding site, a set of independent random numbers, $\varsigma_{i}$, that are uniformly distributed over [0, 1] is generated. Then, the normalized reaction rate at each site is $\lambda_{i}=k_{\mathrm{off}}/k_{\mathrm{on}}^{0}$ for closed bonds and $\lambda_{i}=k_{\mathrm{on}}/k_{\mathrm{on}}^{0}$ for open bonds. The incremental reaction time can be defined as $dt=\frac{1}{k_{\mathrm{on}}^{0}}\min\left\{-\ln\frac{\varsigma_{i}}{\lambda_{i}}\right\}$. The reaction site inducing the incremental reaction time is recorded. The bond state at that site is changed from closed to open or from open to closed. Repeating the above procedure yields the evolution of closed bond density. The mean value of bond number or density can be determined by taking the average over 10,000 different simulation trajectories.

While calculating the evolution of closed bond density, as shown in Figure 7, we take the total number of reaction sites as 140 corresponding to the total bond density of $\xi_{\mathrm{all}}=140{\;\mu m}^{-2}$.

## References

[1] Anderson D.C., Springer T.A. (1987). Leukocyte adhesion deficiency: An inherited defect in the Mac-1, LFA-1, and p150, 95 glycoproteins. Ann. Rev. Med..

[2] Yeung L., Hickey M.J., Wrigh M.D. (2018). The many and varied roles of Tetraspanins in immune cell recruitment and migration. Front. Immunol..

[3] Gay L.J., Felding-Habermann B. (2011). Contribution of platelets to tumour metastasis. Nat. Rev. Cancer.

[4] Eniola A.O., Hammer D.A. (2005). In vitro characterization of leukocyte mimetic for targeting therapeutics to the endothelium using two receptors. Biomaterials.

[5] Muzykantov M.D., Silvia M. (2011). Targeting delivery of drugs in the vascular system. Int. J. Transp. Phenom..

[6] Dembo M., Torney D.C., Saxman K., Hammer D.A. (1988). The reaction-limited kinetics of membrane-to-surface adhesion and detachment. Proc. R. Soc. B-Biol..

[7] Bell G.I., Dembo M., Bongrand P. (1984). Cell adhesion. Competition between nonspecific repulsion and specific bonding. Biophys. J..

[8] Bell G.I. (1978). Models for the specific adhesion of cells to cells. Science.

[9] Marshall B.T., Long M., Piper J.W., Yago T., McEver R.C., Zhu C. (2003). Direct observation of catch bonds involving cell-adhesion molecules. Nature.

[10] Isberg R.R., Barnes P. (2002). Dancing with the host: Flow-dependent bacterial adhesion. Cell.

[11] Evans E., Leung A., Heinrich V., Zhu C. (2004). Mechanical switching and coupling between two dissociation pathways in a P-selectin adhesion bond. Proc. Natl. Acad. Sci. USA.

[12] Bullerjahn J.T., Kroy K. (2016). Analytical catch-slip bond model for arbitrary forces and loading rates. Phys. Rev. E.

[13] Pereverzev Y.V., Prezhdo O.V., Forero M., Sokurenko E.V., Thomas W.E. (2005). The two-pathway model for the catch-slip transition in biological adhesion. Biophys. J..

[14] Xu G.K., Li B., Feng X.Q., Gao H.J. (2016). A tensegrity model of cell reorientation on cyclically stretched substrates. Biophys. J..

[15] Li L., Yao H.M., Wang J.Z. (2016). Dynamic strength of molecular bond clusters under displacement-and force-controlled loading conditions. J. Appl. Mech. Trans..

[16] Chen B., Kemkemer R., Deibler M., Spatz J., Gao H.J. (2012). Cyclic stretch induces cell reorientation on substrates by destabilizing catch bonds in focal adhesions. PLoS ONE.

[17] Zhang Y., Sun G.Y., Lu S.Q., Li N., Long M. (2008). Low spring constant regulates P-selectin-PSGL-1 bond rupture. Biophys. J..

[18] Engler A.J., Sen S., Sweeney H.L., Discher D.E. (2006). Matrix elasticity directs stem cell lineage specification. Cell.

[19] Qian J., Wang J.Z., Gao H.J. (2008). Lifetime and strength of adhesive molecular bond clusters between elastic media. Langmuir.

[20] Qian J., Wang J.Z., Lin Y., Gao H.J. (2015). Lifetime and strength of periodic bond clusters between elastic media under inclined loading. Biophys. J..

[21] Wang J.Z., Gao H.J. (2008). Clustering instability in adhesive contact between elastic solids via diffusive molecular bonds. J. Mech. Phys. Solids.

[22] Zhang W.L., Lin Y., Qian J., Chen W.Q., Gao H.J. (2013). Tuning molecular adhesion via material anisotropy. Adv. Funct. Mater..

[23] Chaudhuri O., Gu L., Darnell M., Klumpers D., Bencherif S.A., Weaver J.C., Huebsch N., Mooney D.J. (2015). Substrate stress relaxation regulates cell spreading. Nat. Commun..

[24] Li L., Zhang W.Y., Wang J.Z. (2016). A viscoelastic–stochastic model of the effects of cytoskeleton remodelling on cell adhesion. R. Soc. Open Sci..

[25] He K.C., Li L., Wang J.Z. (2019). Diffusive–Stochastic–Viscoelastic model for specific adhesion of viscoelastic solids via molecular bonds. Acta Mech. Sin..

[26] Bihr T., Seifert U., Smith A.S. (2015). Multiscale approaches to protein-mediated interactions between membranes—Relating microscopic and macroscopic dynamics in radially growing adhesions. New J. Phys..

[27] Fenz S.F., Bihr T., Schmidt D., Merkel R., Seifert U., Sengupta K., Smith A.S. (2017). Membrane fluctuations mediate lateral interaction between cadherin bonds. Nat. Phys..

[28] Krobath H., Różycki B., Lipowsky R., Weikl T.R. (2009). Binding cooperativity of membrane adhesion receptors. Soft Matter.

[29] Rozycki B., Lipowsky R., Weikl T.R. (2010). Segregation of receptor–ligand complexes in cell adhesion zones: Phase diagrams and the role of thermal membrane roughness. New J. Phys..

[30] Li N., Lu S.Q., Zhang Y., Long M. (2015). Mechanokinetics of receptor–ligand interactions in cell adhesion. Acta Mech. Sin..

[31] Robin T., Sokolov I.M., Urbakh M. (2018). Life time of catch bond clusters. Phys. A.

[32] Wang J.Z., Gao H.J. (2010). Size and shape dependent steady-state pull-off force in molecular adhesion between soft elastic materials. Int. J. Fract..

[33] Chen B., Gao H.J. (2010). Mechanical principle of enhancing cell-substrate adhesion via pre-tension in the cytoskeleton. Biophys. J..

[34] He S.J., Su Y.W., Ji B.H., Gao H.J. (2014). Some basic questions on mechanosensing in cell–substrate interaction. J. Mech. Phys. Solids.

[35] Li L., Tang H., Wang J.Z., Lin J., Yao H.M. (2018). Rolling adhesion of cell in shear flow: A theoretical model. J. Mech. Phys. Solids.

[36] Du Y., Peng S., Cui Y.H., Lv S.Q., Zhang Y., Long M. (2015). Combined modeling of cell aggregation and adhesion mediated by receptor–ligand interactions under shear flow. Theor. Appl. Mech. Lett..

[37] Long M., Goldsmith H.L., Tees D.F.J., Zhu C. (1999). Probabilistic modeling of shear-induced formation and breakage of doublets cross-linked by receptor-ligand bonds. Biophys. J..

[38] Dong C., Lei X.X. (2000). Biomechanics of cell rolling: Shear flow, cell-surface adhesion, and cell deformability. J. Biomech..

[39] Reboux S., Richardson G., Jensen O.E. (2007). Bond tilting and sliding friction in a model of cell adhesion. Proc. R. Soc. A-Math. Phys. Eng. Sci..

[40] Xu X.F., Efremov A.K., Li A., Lai L.P., Dao M., Lim C.T., Cao J.S. (2013). Probing the cytoadherence of malaria infected red blood cells under flow. PLoS ONE.

[41] Sircar S., Bortz D.M. (2013). Impact of flow on ligand-mediated bacterial flocculation. Math. Biosci..

[42] Wei M.J., Zhang R.B., Zhang F., Zhang Y.C., Li G.X., Miao R.J., Shao S.H. (2019). An Evaluation Approach of Cell Viability Based on Cell Detachment Assay in a Single-Channel Integrated Microfluidic Chip. ACS Sens..

[43] Tan J.F., Ding Z.Y., Hood M., Li W. (2019). Simulation of circulating tumor cell transport and adhesion in cell suspensions in microfluidic devices. Biomicrofluidics.

[44] Gong X.B., Li Y.Q., Gao Q.C., Cheng B.B., Shen B.R., Yan Z.Q., Jiang Z.L. (2011). Adhesion behavior of endothelial progenitor cells to endothelial cells in simple shear flow. Acta Mech. Sin..

[45] Efremov A., Cao J.S. (2011). Bistability of cell adhesion in shear flow. Biophys. J..

[46] Yago T., Wu J.H., Wey C.D., Klopocki A.G., Zhu C., McEver R.P. (2004). Catch bonds govern adhesion through L-selectin at threshold shear. J. Cell Biol..

[47] Sarangapani K.K., Yago T., Klopocki A.G., Lawrence M.B., Fieger C.B., Rosen S.D., McEver R.P., Zhu C. (2004). Low force decelerates L-selectin dissociation from P-selectin glycoprotein ligand-1 and endoglycan. J. Biol. Chem..

[48] Alon R., Chen S.Q., Puri K.D., Finger E.B., Springer T.A. (1997). The kinetics of L-selectin tethers and the mechanics of selectin-mediated rolling. J. Cell Biol..

[49] Beste M.T., Hammer D.A. (2008). Selectin catch–slip kinetics encode shear threshold adhesive behavior of rolling leukocytes. Proc. Natl. Acad. Sci. USA.

[50] Caputo K.E., Lee D., King M.R., Hammer D.A. (2007). Adhesive dynamics simulations of the shear threshold effect for leukocytes. Biophys. J..

[51] Whitfield M., Ghose T., Thomas W. (2010). Shear-Stabilized Rolling Behavior of E. Coli Examined with Simulations. Biophys. J..

[52] Kampen V., Godfried N. (1981). Stochastic Processes in Physics and Chemistry.

[53] Erdmann T., Schwarz U.S. (2007). Impact of receptor-ligand distance on adhesion cluster stability. Eur. Phys. J. E.

[54] Polizzi N.F., Therien M.J., Beratan D.N. (2016). Mean First-Passage Times in Biology. Isr. J. Chem..

[55] Erdmann T., Schwarz U.S. (2004). Stability of adhesion clusters under constant force. Phys. Rev. Lett..

[56] Schubert G. (1967). Viscous flow near a cusped corner. J. Fluid Mech..

[57] Yao H.M. (2013). A generalized model for adhesive contact between a rigid cylinder and a transversely isotropic substrate. J. Appl. Mech..

[58] Lawrence M.B., Springer T.A. (1991). Leukocytes roll on a selectin at physiologic flow rates: Distinction from and prerequisite for adhesion through integrins. Cell.

[59] Schmid-Schoenbein G.W., Fung Y.C., Zweifach B.W. (1975). Vascular endothelium-leukocyte interaction; sticking shear force in venules. Circ. Res..

[60] Grover W.H., Bryan A.K., Diez-Silva M., Suresh S., Higgins J.M., Manalis S.R. (2011). Measuring single-cell density. Proc. Natl. Acad. Sci. USA.

[61] Evans E.A., Calderwood D.A. (2007). Forces and bond dynamics in cell adhesion. Science.

[62] Kong F., Garcia A.J., Mould A.P., Humphries M.J., Zhu C. (2009). Demonstration of catch bonds between an integrin and its ligand. J. Cell Biol..

[63] Popescu G., Auerbach A. (2003). Modal gating of NMDA receptors and the shape of their synaptic response. Nat. Neurosci..

[64] Sun L., Cheng Q.H., Gao H.J., Zhang Y.W. (2012). Effect of loading conditions on the dissociation behaviour of catch bond clusters. J. R. Soc. Interface.

[65] Rakshit S., Zhang Y.X., Manibog K., Shafraz O., Sivasankar S. (2012). Ideal, catch, and slip bonds in cadherin adhesion. Proc. Natl. Acad. Sci. USA.

[66] Chang K., Tees D.F., Hammer D.A. (2000). The state diagram for cell adhesion under flow: Leukocyte rolling and firm adhesion. Proc. Natl. Acad. Sci. USA.

[67] Puri K.D., Chen S.Q., Springer T.A. (1998). Modifying the mechanical property and shear threshold of L-selectin adhesion independently of equilibrium properties. Nature.

[68] Zhu C., Yago T., Lou J.Z., Zarnitsyna V.I., McEver R.P. (2008). Mechanisms for flow-enhanced cell adhesion. Ann. Biomed. Eng..

[69] Finger E.B., Puri K.D., Alon R., Lawrence M.B., von Andrian U.H., Springer T.A. (1996). Adhesion through L-selectin requires a threshold hydrodynamic shear. Nature.

[70] Alon R., Hammer D.A., Springer T.A. (1995). Lifetime of the P-selectin-carbohydrate bond and its response to tensile force in hydrodynamic flow. Nature.

[71] Thomas W.E., Trintchina E., Forero M. (2002). Bacterial adhesion to target cells enhanced by shear force. Cell.

[72] Gillespie D.T. (1976). A general method for numerically simulating the stochastic time evolution of coupled chemical reactions. J. Comput. Phys..

[73] Gillespie D.T. (1977). Exact stochastic simulation of coupled chemical reactions. J. Phys. Chem. C.

